# Speech recognition with hearing aids for 10 standard audiograms: English version

**DOI:** 10.1007/s00106-020-00843-y

**Published:** 2020-03-24

**Authors:** C. Dörfler, T. Hocke, A. Hast, U. Hoppe

**Affiliations:** 1 Audiology Department and CI Centre CICERO Ear, Nose and Throat Clinic, University Hospital Erlangen, Waldstraße 1, 91054 Erlangen, Germany; 2 Cochlear Deutschland GmbH & Co. KG, Hannover, Germany

**Keywords:** Maximum word recognition score, Speech audiometry, Tone audiometry, Speech comprehensibility, IEC 60118-15

## Abstract

**Background:**

Improvement of speech perception in quiet is an important goal of hearing aid provision. In practice, results are highly variable. The aim of this study was to investigate the relationship between type and extent of hearing loss (audiogram type), maximum word recognition score, and aided speech perception.

**Materials and methods:**

Pure tone and speech audiometric data of 740 ears in 370 patients were reviewed. All subjects visited our hearing center for hearing aid evaluation between 2012 and 2017. The maximum word recognition score (WRS_max_) and the monosyllabic speech recognition score with hearing aids, WRS_65_(HA) were analyzed for 10 different standard audiogram types.

**Results:**

The WRS_65_(HA) with hearing aids for different degrees of hearing loss is, within error boundaries, comparable to previous investigations and shows a difference of 10–20 percentage points to the WRS_max_. This difference tends to be larger for flat and moderately sloping audiograms compared to steep-sloping audiograms. The ratio WRS_65_(HA)/WRS_max_ can be interpreted as an efficiency factor for hearing aid provision, since it relates speech recognition with hearing aids to the maximally achievable information carrying capacity of the hearing impaired.

**Conclusion:**

The expectation regarding hearing aid provision has to be adjusted according to maximum word recognition score, the derived quality measures, degree of hearing loss, and audiogram type.

## Introduction

Hearing loss is one of the five most frequent disorders with a substantial impact on quality of life [17]. In Germany, an estimated 10–12 million adults suffer from sensorineural hearing loss that requires treatment [23, 25] without any causal therapy available. As a rule, a hearing aid (HA) is indicated in such cases [24]. Here, the primary aim is an improvement of speech recognition in quiet and, in particular, in noise [5, 13]. In addition to the indication, an evaluation of the hearing aid by an ear, nose, and throat (ENT) physician is also vitally important [10, 14, 23].

A previous study [7] investigated the relationship between unaided audiometric measures and speech recognition with hearing aids. Speech recognition in quiet was adopted as a surrogate parameter for success of the hearing aid provided. Speech recognition depends on hearing loss, hearing-aid fitting and age, among other factors [21]. It was also shown that the hearing-aided (HA) monosyllable recognition (word recognition score, WRS) at 65 dB sound pressure level (SPL), WRS_65_(HA), for moderate and severe hearing loss is on average 20 percentage points below the maximum word recognition score (WRS_max_), as measured in the speech audiogram with headphones. For a sample of *n* = 181 individuals provided with hearing aids, a statistical correlation was found between the mean of the hearing thresholds at 0.5, 1, 2 and 4 kHz (four-frequency pure-tone average, 4FPTA) and the WRS_max_ or WRS_65_(HA) was found. This can be described by the following equations.

1 $$\mathrm{WRS}_{\max }[\% ]=100\cdot \frac{e^{\left(\beta _{0}+\beta _{1}\cdot 4FPTA\right)}}{1+e^{\left(\beta _{0}+\beta _{1}\cdot 4FPTA\right)}}$$

with $$\beta _{0}=5.99\pm 0.08$$ and $$\beta _{1}=-0.0793\pm 0.0012$$

2 $$\mathrm{WRS}_{65}\left(HA\right)[\% ]=100\cdot \frac{e^{\left(\beta _{0}+\beta _{1}\cdot 4FPTA\right)}}{1+e^{\left(\beta _{0}+\beta _{1}\cdot 4FPTA\right)}}$$

with $$\beta _{0}=3.96\pm 0.06$$ and $$\beta _{1}=-0.0673\pm 0.0011$$

**Fig. 1:**
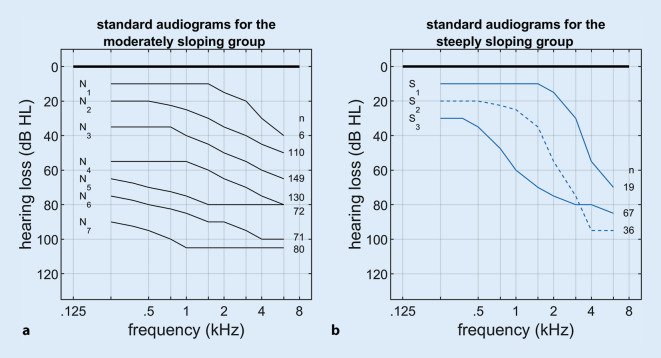
Classification of pure-tone audiograms according to Bisgaard [1]. **a** Flat to moderately sloping audiograms. **b** Steeply sloping audiograms. The corresponding types (N_1_–N_7_ or S_1_–S_3_) are shown to the *left* of the audiograms. To the *right* of the curves, the respective numbers of cases investigated here are noted

In other studies with hearing aid users, comparable values were found for the parameters $\beta _{0} \mathrm{and}\:\beta _{1}$[12, 21]. For further analysis regarding the frequency-dependent course of hearing loss, the proposed classification into 10 standard audiograms by Bisgaard et al. [1] seems appropriate. This classification is based on a statistical analysis of 28,244 hearing-threshold measurements; it was motivated by the requirements of German Institute for Norms (*Deutsches Institut für Normung*, DIN) EN 60118-15 and is intended to represent typical audiograms of hearing aid users. This DIN norm requires a realistic characterization of the hearing aids and the hearing aid setting. It therefore makes sense to apply these standard audiograms to clinical issues associated with hearing aid provision. All standard audiograms shown in Fig. 1 show increasing hearing loss with increasing frequency. They have been classified into those with low to medium (*N*-type, with a maximum decay of 20 dB per octave) and with strong (S-type with maximum decay of at least 25 dB per octave) frequency dependence. These types were subdivided into seven or three subtypes depending on the degree of hearing loss (N_1_–N_7_ and S_1_–S_3_).

The aim of the present study was to investigate speech recognition with and without a hearing aid as a function of the extent and the frequency course of hearing loss for a large population of hearing aid users. In addition, the authors investigated the extent to which the earlier findings [7] also apply to a present-day population with modern hearing systems.

## Patients and methods

### Patients

In this retrospective study, 2357 hearing aid examinations were evaluated; these were performed between August 2012 and September 2017 in the Erlangen ENT Clinic, Germany. Bilateral hearing aid users with at least 3 months of hearing aid experience, German as mother tongue and a minimum age of 18 years were included. The exclusion criteria included abnormal otoscopic results, a mean air–bone gap at 0.5, 1, 2 and 4 kHz of more than 5 dB, an asymmetry of 4FPTA on both sides of more than 20 dB and any technical defects in the hearing aids. The data from a total of 740 ears from 370 users (182 men, 188 women) aged 21 to–98 years (mean, 62.8 years; standard deviation, 16.2 years) remained.

### Measurements

Pure-tone air-conduction thresholds were measured for frequencies between 0.125 and 8 kHz and bone-conduction thresholds between 0.25 and 6 kHz. The Freiburg monosyllable test was used to measure speech recognition. The measurements in quiet were performed with monaural presentation using headphones, initially at 65 dB SPL (WRS_65_).

Subsequently, the presentation level was increased in increments of 5–15 dB until 100% speech intelligibility was attained (unless the sound level became intolerable for the user or the audiometer limit of 120 dB SPL was reached). The uncomfortable loudness level (UCL) corresponds to the lowest speech presentation level that is no longer tolerated. In addition to WRS_65_, WRS_max_ was recorded.

The hearing aid check included a visual inspection and feedback provocation of oscillation. In addition, qualified personnel (hearing aid acousticians) checked whether the type and model of hearing aid provided were appropriate for the individual’s hearing loss. The amplification was checked by measurement in situ. The speech test with hearing aid was conducted in free field at 65 dB_SPL_ for the left and right ear separately; the contralateral side was adequately blocked with earplugs.

### Data analysis

For all patients, the audiometric data of the 740 ears were analyzed for each side separately. Cases were classified according to their hearing-loss characteristics into the 10 audiogram types N_1_–N_7_ and S_1_–S_3_ according to Bisgaard [1] by using the minimum Euclidean distance.

For comparison of WRS_max_ and WRS_65_(HA) the difference

3 $$D=mEV-EV_{65}\left(HG\right)$$

and the quotient

4 $$Q=\frac{EV_{65}\left(HG\right)}{mEV}$$

were calculated for both quantities.

The quotient Q stands for the proportion of the WRS_max_ that can be amplified by the hearing aid to give a speech perception of 65 dB SPL. It can be interpreted as the speech-audiometric efficiency factor of the hearing aid in question.

Logistical regression analysis of speech recognition, WRS_max_ or WRS_65_(HA), as a function of hearing loss, 4FPTA, was performed in a manner analogous to that described by Hoppe et al. [7]. The non-parametric Jonckheere–Terpstra test was used to test the group trends within the two basic types of audiogram [4]. The statistical tests were carried out with SPSS V24 (IBM, Armonk, NY, USA), and the images were generated with Matlab® R2017a (Mathworks, Natick/MA, USA).

## Results

### Classification of Audiograms

The 10 standard audiogram types, according to Bisgaard [1], are shown in Fig. 1. The case numbers (*n*) are shown to the right of the curves.

Table 1 summarizes the group sizes and statistical measures for age and 4FPTA for each type of audiogram after classification of the 740 cases. The mean age in the individual groups varied from 50 years to 68 years.

**Table 1:** Audiogram classification, group sizes, four-frequency pure-tone average (4FPTA) and age

Audiogram classification and group size		4FPTA (dB HL)	Age (years) (mean±SD)
N_1_	6	19 ± 4	58 ± 4
N_2_	110	34 ± 4	65 ± 11
N_3_	149	47 ± 4	65 ± 15
N_4_	130	61 ± 4	66 ± 16
N_5_	72	74 ± 6	62 ± 18
N_6_	71	89 ± 5	60 ± 16
N_7_	80	109 ± 8	50 ± 18
S_1_	19	26 ± 4	62 ± 23
S_2_	36	46 ± 7	68 ± 13
S_3_	67	66 ± 7	65 ± 15

*SD* standard deviation

**Fig. 2:**
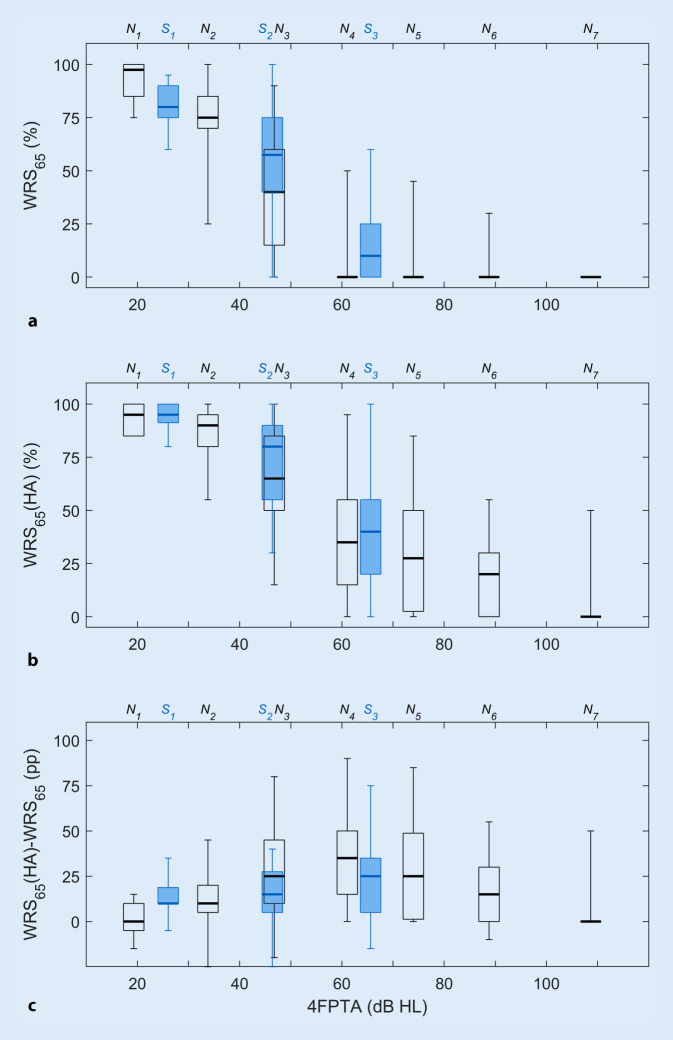
Monaural speech recognition for the 10 audiogram types (*black*, N‑type; *blue*, S‑type), **a** without and **b** with hearing aid depending on four-frequency pure-tone average (*4FPTA*). The corresponding differences (percentage points) are shown in (**c**). The measurements without a hearing aid were made with headphones at 65 dB sound pressure level (SPL) and the hearing aid measurements were made in free field at 65 dB SPL. Each box plot shows the median, first/third quartiles, and minimum/maximum. The x‑position of a box plot corresponds to the mean 4FPTA of the cases that were assigned to the corresponding audiogram type (as listed in Table 1)

Fig. 2 shows the monaural speech recognition by the hearing aid users as box plots in relation to 4FPTA and audiogram type. The upper part (a) shows the results of the measurement with headphones at 65 dB SPL, while the middle part (b) shows the values measured in free field with hearing aids at 65 dB SPL. The lower part (c) shows the benefit from hearing aid provision as the difference between the two measurements. The data are presented for each audiogram type separately, with black box plots for N‑type and blue box plots for S‑type hearing loss.

For mild hearing loss (N_1_, N_2_ and S_1_) all hearing aid users achieved a WRS_65_(HA) greater than 50%. For moderate hearing loss (N_3_ and S_2_), this was achieved in 75% of the cases. Of the users with severe hearing loss—groups N_4_ and N_5_, as well as S_3_—only a quarter had speech recognition scores above 50%. For the groups N_6_ and N_7_, with profound hearing loss up to deafness, this 50% score was observed in only a few isolated cases: four out of 71 in the N_6_ group and one out of 80 in the N_7_ group. As shown in Fig. 2c, users with hearing losses between 40 and 90 dB showed the greatest median improvements on provision of a hearing aid. However, 5.5% of the cases show reduced speech understanding in the free-field measurement with a hearing aid compared with the headphone measurement. This reduction was between 5 and 25 percentage points.

**Fig. 3:**
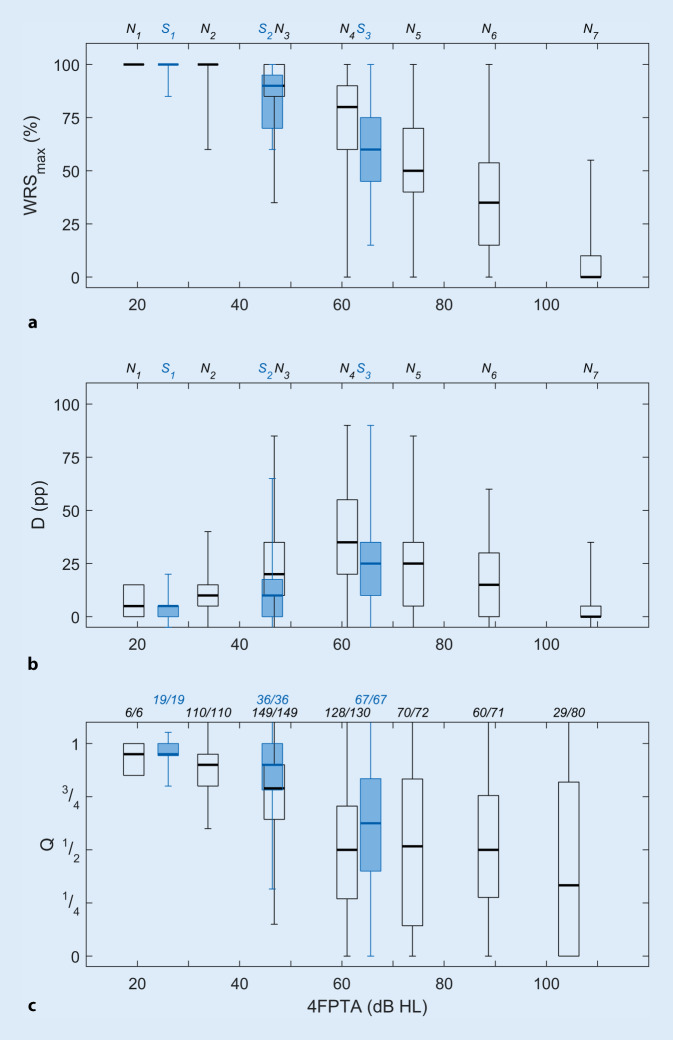
Maximum monosyllable recognition (*WRS*_*max*_) (**a**) and the relation to speech recognition (**b**,**c**) with hearing aid for the 10 types of audiogram (*black*, N‑type; *blue*, S‑type) depending on four-frequency pure-tone average (*4FPTA*); **b** shows the difference (percentage points) and **c** the ratio of the two measurements. The WRS_max_ was measured with headphones and the hearing aid measurement in free field at 65 dB sound pressure level. The assignment of the box plots to the types of audiogram shown in Fig. 1 is shown at the *top*. Each box plot shows the median, first/third quartiles and minimum/maximum. The x‑position of a box plot corresponds to the mean 4FPTA of the cases that were assigned to the corresponding audiogram type. In the *lower part* (**c**), only those cases with WRS_max_ >0% can be displayed. The proportions of these cases in groups N_1–7_ and S_1–3_ are each given above the box plot in (**c**)

Fig. 3 shows the correlation between the measured maximum word recognition score and the scores attained with a hearing aid. The WRS_max_ for the individual groups is shown in Fig. 3a. Fig. 3b,c represent the difference D and the quotient Q, respectively, for each audiogram type. For values of D near zero and Q near unity the WRS_max_ is approximated by the WRS_65_(HA). Fig. 3a shows the reduction in maximum monosyllable recognition associated with increasing hearing loss. The differences shown in Fig. 3b reveal a similar picture, showing saturation effects of the test, as in Fig. 2c. The calculation of the quotients Q is only possible for cases with WRS_max_ greater than zero. For example, for the N_6_-type group, WRS_max_ above zero was found in 60 of 71 cases. The corresponding results for the other groups are stated above the border of Fig. 3c. For the analysis of the influence of the audiogram type on Q, the two basic types (N-type and S‑type) were considered separately. The trend analysis for the medians was performed with the Jonckheere–Terpstra test. This results in a significant dependence of the efficiency Q on the extent of hearing loss for both basic types. The test statistic *J*, the standardized test statistic *z* and the corresponding *p* value are: *J* = 36468, *z* = −11.7 and *p* < 0.001 for N‑type audiograms and *J* = 1020, *z* = −5.8 and *p* < 0.001 for S‑type audiograms.

**Fig. 4:**
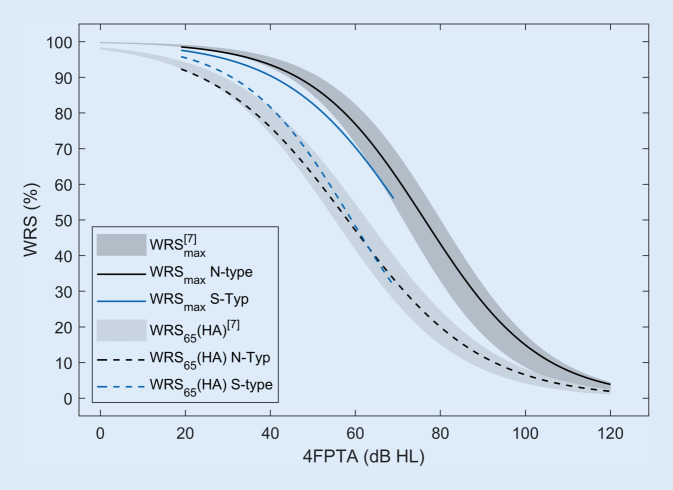
Logistical regression of monosyllable recognition (*WRS*), maximum WRS (*WRS*_*max*_) or hearing-aided WRS at 65 dB (*WRS*_*65*_[*HA*]) as a function of hearing loss, four-frequency pure-tone average (*4FPTA*), for N and S‑type audiograms according to Bisgaard et al. [1]. The *grey areas* represent the 95% confidence interval of the logistical regression according to Hoppe et al. [7]. The *black* (*blue*) *lines* are the regression curves of the present data for the N‑type (S-type) audiograms

Fig. 4 displays the present results in relation to earlier data from 2011 and 2012 [7]. The grey areas in the figure represent the 95% confidence interval of the logistical regression of the reference data [7]: dark grey for WRS_max_ and light grey for WRS_65_(HA). The results of the logistical regression of the WRS_max_ or WRS_65_(HA) are shown separately for N‑type and S‑type hearing-loss audiograms. The regression calculation was performed for 618 *N*-type cases (ears) and for 122 S‑type cases. While the curves for WRS_max_ of the N‑type groups lie within the 95% confidence interval, the fit of WRS_max_ for the S‑type groups lies below the 95% confidence.

A different picture emerges for speech recognition with hearing aids: The two regression functions for the WRS_65_(HA) are largely within the 95% confidence interval, with a tendency toward better values for the S‑type groups (severe hearing loss) compared with N‑type groups (mild to moderate hearing loss).

## Discussion

The aim of this work was to investigate the correlation between the pure-tone and speech-audiometric measures as assessed during hearing aid evaluation. The focus was on: (i) understanding speech in quiet, (ii) the improvement through a hearing aid as the principal effect of hearing-aid provision and (iii) the relationship to maximum monosyllable recognition. Extending the scope of previous work [7, 8], the cases were classified, on the basis of audiogram progression, according to the various audiogram types defined by Bisgaard [1]. For the same 4FPTA, monosyllabic word recognition with a hearing aid is significantly better for users with highly frequency-dependent hearing loss than for those whose audiograms show only moderate frequency dependence.

### Specific audiogram patterns and speech recognition

WRS_65_, with or without a hearing aid, is primarily determined by the 4FPTA. For the WRS_65_(HA), the type of audiogram provides additional information (see Fig. 2b). WRS_65_(HA) decays monotonically, in both N and S audiogram types, with increasing 4FPTA. However, the regression function for the hearing aids in patients with S‑type audiograms is above the regression function for those with N‑type audiograms. In particular, a comparison of the N_3_ and S_2_ groups—with nearly equal 4FPTA and approximately equal age distribution—shows that the WRS_65_(HA) for the S‑type audiograms is 15 percentage points above the WRS_65_(HA) for the N‑type audiograms. The results of the logistical regression support this finding: In low to moderate hearing loss, good speech recognition can be achieved with hearing aids, especially in cases where the audiogram is steep. While in the S groups the steep drop in the audiogram in the headphone measurement of WRS_max_ obviously leads to poorer results, the provision of a hearing aid compensates better for the frequency-specific attenuation component of hearing loss.

In the N groups, the frequency-independent amplification in the WRS_max_ measurement thus corresponds more closely to the hearing-aid fitting than in the S groups, where low frequencies need no or only small amplification. This partly explains the differences in the closeness of approach of WRS_65_(HA) to WRS_max_, as shown in Fig. 3c.

A further explanation could be the increasing availability of open-fit hearing aids in the last few years, for which the S‑type audiograms are well suited in respect of the tonal-audiometric prerequisites [11]. An initially lower WRS_max_ of the S group can be compensated for by the advantages associated with the open-fit design [3, 20].

### Comparison with previous investigations

Fig. 4 shows the results of the logistical regression for the N and S types. For specific 4FPTA regions, differences can be detected for the N‑ and S‑type audiograms. However, in view of the far greater individual variation, the differences found in the regression are unlikely to entail major clinical consequences. Consequently, the reduction of the pure-tone hearing loss in 4FPTA seems to appropriately describe the description of the correlations with the measurements of recognition, at least for large groups of patients and the inferences drawn from their data [7, 8, 12, 15, 21]. The comparison of the aided speech recognition shown in Fig. 4 with that in an earlier comparable population analysis for January 2011 to July 2012 [7] shows differences in the range of a few percentage points. Accordingly, no differences were observed for monosyllable recognition between the results of that study and the present results. This finding is consistent with that of a recent study [2] on subjective hearing quality.

### Practical aspects of hearing aid evaluation using speech audiometry

Another aspect of the present work was the further description of the relationship between WRS_max_ and WRS_65_(HA) against the background of the speech audiometric evaluation of a hearing aid provision. The Guideline for Hearing Aids of the German Joint Federal Committee (*Gemeinsamer Bundesausschuss*) [5] requires an increase of WRS_65_(HA) over WRS_65_ of at least 20 percentage points. It also calls for WRS_65_(HA) to converge with the WRS_max_ as closely as possible, even if this is often not achieved [7, 8, 12, 15, 21]. The main reasons for this may be a lack of acceptance of the required acoustic amplification, overly low residual dynamics, reduced adaptation to the aid provided in elderly users or technical causes [3, 7, 21, 26]. Only for hearing losses below 40 dB and above 100 dB are the differences between WRS_max_ and WRS_65_(HA) below 20 percentage points for the majority of hearing aids provided. For these areas, the known floor and ceiling effects of the language test used will necessarily limit the difference, and thus its diagnostic value.

For mild to moderate hearing loss, most hearing aids can achieve an efficiency factor greater than 3/4. From a moderate degree of hearing loss upwards (4FPTA >60 dB) this falls to 1/2. The fact that the efficiency factor is not defined for the case of WRS_max_ = 0% is of little practical relevance here. In these cases alternative speech tests should be considered. If this is not possible, then speech audiometry is in any case not an appropriate method for assessing hearing aid provision.

Table 2 illustrates the significance of the efficiency factor Q on the basis of four examples with identical differences: If the hearing aid achieves the maximum word recognition score, WRS_max_ and WRS_65_(HA) are identical. The difference is zero and the efficiency factor one. Relevant differences only arise if the WRS_max_ is not achieved. The efficiency Q quantifies the actually attained proportion of the greatest possible speech recognition (information-carrying capacity [6, 9]) and can be used, in addition to the difference, for the assessment of a hearing aid.

**Table 2:** Speech-audiometric parameters for four cases in which the monosyllable recognition with hearing aid (WRS_65_[HA])was 10 percentage points below the maximum word recognition score (WRS_max_). In these cases, the speech-audiometric efficiency factor Q varied between 0 and 0.9

Example	WRS_max_	WRS_65_(HA)	D	Q
#1	100	90	10	0.9
#2	50	40	10	0.8
#3	25	15	10	0.6
#4	10	0	10	0

The results presented here, obtained at a specialized ENT clinic with an associated hearing-aid and cochlear-implant centre, are confirmed by other work [12, 15, 18, 19, 21] at similar centres. Nonetheless, there remains some uncertainty as to whether these results are representative of the overall hearing-aid provision to a wider user spectrum. Therefore, it would also be desirable to conduct user-oriented studies, outside large hearing centres, on the current state of hearing aid provision. To assess the comparability of results, these tests should adhere to speech-audiometric standards as proposed for clinical studies on hearing improvement [22], for example with active middle-ear implants [16]. These should also include, as far as possible, speech-intelligibility measurements in noise.

## Practical conclusion

- The ratio between speech understanding with the hearing aid, WRS_65_(HA), and maximum word recognition score, WRS_max_ measured with headphones, provides additional information on the speech-audiometric efficacy of the hearing aid.
- The efficiency of a hearing aid provision is on average higher with strongly frequency-dependent S-type audiograms than with the flatter N-type audiograms.
- For hearing losses below 60 dB HL, the efficiency is close to one; above 60 dB HL, on average, values of only 1/2 are achieved.
